# Analysis of long-term observations of the large group of Russian patients with Hunter syndrome (mucopolysaccharidosis type II)

**DOI:** 10.1186/s12920-021-00922-1

**Published:** 2021-03-06

**Authors:** Alla Nikolaevna Semyachkina, Elena Yurievna Voskoboeva, Ekaterina Alexandrovna Nikolaeva, Ekaterina Yurievna Zakharova

**Affiliations:** 1 Department of Clinical Genetics, Research and Clinical Institute of Pediatrics Named After Yuri Veltischev of the Pirogov Russian National Research Medical University of the Russian Ministry of Health, 2 Taldomskaya St., Moscow, 125412 Russia; 2 Research Centre for Medical Genetics RAN, 1 Moskvorechie St., Moscow, 115522 Russia

**Keywords:** Mucopolysaccharidosis, Hunter syndrome, Clinical and genetic analysis, Social adaptation

## Abstract

**Background:**

This article presents the results of long-term observations and comparative analysis of genotype–phenotype features in a large group of patients (227 males and one female) with a severe, intermediate and mild form of Hunter syndrome, evaluating the quality and span of their lives, as well as their ability to social adaptation.

**Methods:**

We used electrophoresis of glycosaminoglycans of urine, determination of the activity of lysosomal enzymes in plasma, in dried blood spots according to the generally accepted method and DNA analysis.

**Results:**

The clinical symptomatology of 228 patients with Hunter syndrome was characterized by growth retardation, lesions of the bronchopulmonary, cardiovascular, nervous systems, etc. Thirty-five patients had an attenuated form of the disease. DNA was available from all patients. 19 patients from 10 families had a mild form of the disease. 42 patients from 41 families had an intermediate form of the disease. All other patients had a severe form of the disease. We provide brief clinical examples of some patients with a mild form of Hunter syndrome. Currently, 113 patients with Hunter syndrome receive enzyme replacement therapy (idursulfase or idursulfase beta).

**Conclusion:**

The long-term study of the large number of patients with Hunter syndrome helped identify disease-associated variants leading to severe and mild forms of the disease. The treatment effect and successful social adaptation of patients with a mild form of Hunter syndrome were revealed.

## Background

Hunter syndrome or mucopolysaccharidosis type II (MPS II) is a rare disease with frequency ranges from 1:100,000 to 1:170,000 newborn boys [1–4].

Hunter syndrome is the only type of mucopolysaccharidoses that inherited as X-linked recessive trait. Hence, the majority of patients with Hunter syndrome are male. However, few cases of the disease in girls were described. Most of them are associated with structural abnormalities, inactivation disorders, or monosomy of chromosome X [5–7].

The clinical symptoms of Hunter syndrome usually become noticeable during the first two years of life. The disease is characterized by the progressive course. Clinical manifestations include rough facial features, sunken nose, full lips, hypertelorism, large tongue, corneal clouding, megalocephaly, thick coarse hair, short neck, brachidactyly of the hands and feet, contractures of joints, short stature, diffuse muscle hypotension, hepatosplenomegaly, umbilical and inguinal hernias, cardiomyopathy and gross delay in psycho-speech and motor development for most patients. After the first phenotype descriptions, two clinically different forms of Hunter syndrome were identified: a classical, severe form with severe somatic sings, progressive mental retardation, death at the age of 20 years or earlier; and a mild form characterized by longer life expectancy, fertility and minimal reduced intellect [8]. Later, it was suggested considering MPS II as a continuum between two extremes (severe and attenuated) [9].

The disease is caused by deficiency of the lysosomal enzyme iduronate-2-sulfatase (I2S) [10]. This defect is a result of different nucleotide variants in the *IDS* gene. The *IDS* gene is located on the locus Xq28 and consists of 9 exons [11]. At present, over 600 nucleotide variants in the *IDS* gene have been identified*.* Most of them are point changes (missense or nonsense variants), 28% are minor deletions and insertions, and 9% are splicing substitutions. Gross rearrangements account for 11%, among which 7% are partial or complete deletions of the *IDS* gene [12]*.*

The I2S deficiency leads to the accumulation in different tissues of two types of glycosaminoglycans (GAG), i.e. heparan sulfate and dermatan sulfate resulting in the formation of multisystem pathology [13, 14].

The diagnosis of Hunter syndrome in Russia is carried out in several stages. The first stage is based on the identification and assessment of phenotype signs. The second stage consists of determination of urinary GAG level and of their fractions, primarily heparan and dermatan sulfates. At the third stage, the activity of the lysosomal enzyme I2S is measured in plasma or in dry blood spots. The fourth and the final stage is the molecular genetic analysis of the *IDS* gene.

A prerequisite is also the DNA analysis of the child’s mother to confirm the carrier of the pathogenic variant of the *IDS* gene. In Russia, the results of the final stage are necessary for the reasonable appointment of enzyme replacement therapy to the patient and successful family genetic counseling.

The purpose of this article is to present results of the comparative analysis of genotype–phenotype features in a large group of patients with Hunter syndrome, to assess the quality and duration of their life, as well as the social adaptability of patients with a mild form of the disease. We focused on describing patients with a mild form of disease, which is important for us since there are not many patients with this form of the disease. Moreover, perhaps physicians should be more thoughtful to identify patients for timely treatment.

## Methods

Over the last 30 years (1989–2019), we have observed 228 patients with Hunter syndrome (mucopolysaccharidosis type II): 227 male and one 4-year old girl. The age of patients ranged from two to 65 years. First, all the patients were presumably diagnosed with Hunter syndrome based on the clinical data.

To confirm the disease, we determined the urinary GAG level and measured the I2S activity. As the final step, the DNA analysis of the *IDS* gene was carried out.

We used the following materials and methods:

1. Electrophoresis of urinary GAG.

Extraction of GAG from urine and electrophoresis of urinary GAG was carried out exactly according to a standard method that has been described previously [15].

2. Biochemical assay.

The I2S activity was measured in plasma, as was described in the literature [16]. In brief, to 10 μl 5 × diluted in 0.2%BSA plasma 20 μl substrate (1.25 mM MU-alphaIdoA-2S) was added and the mixture was incubated for 4 h at 37 °C. Then 20 μl PiCi buffer and 10 μl LEBT solution (lysosomal enzymes purified from bovine testis) was added with subsequent incubation for 24 h at 37 °C. After adding 200 μl, stop-buffer fluorescence of MU was read. The normal range of the I2S activity was 297–705 nmol/4h/ml. Since 2019, the I2S activity was measured in dried blood spots by MS/MS methods, using the commercial kit [17]. Measuring was performed according to the manufacturer’s protocol.

3. DNA analysis.

The DNA extraction was carried out according to the manufacturer’s protocol using the DIAtomt DNA Prep100 kit (Isogene Lab. Ltd., Russia). The nine exons and exon–intron boundaries of the *IDS* gene were amplified from DNA samples with primers sets designed according to the reference sequence NC_000023.11. PCR conditions and primers were available upon request. Sequencing was performed according to the manufacturer’s protocol on an ABI Prism 3500XL (Applied Biosystems). To detect gene-pseudogene recombination, two pair primers were used as described in the literature [18]. Gross deletion of the *IDS* gene was detected only on the genomic DNA level. Break-point of the deletion and inversion *IDS/IDS2* were not revealed in this study.

## Results

The clinical symptoms of mucopolysaccharidosis type II in the patients observed are presented in Table 1. The symptoms included growth retardation, lesion of the bronchopulmonary system, heart and blood vessels, central nervous system, and hearing organ.

**Table 1:** Panel of clinical symptoms in patients with Hunter syndrome (n = 228)

Clinical symptoms	Number of criteria, %
Changes in facial features by «gargoylism» type	100
Short stature	95
Skeletal anomalies (dysostosis multiplex)	100
Pathologies of the cardiovascular system	100
Cardiomyopathy	35
Anomalies of the heart valves	100
Narrowing of coronary arteries	10
Rhythm disturbance	20
Obstructive conditions of the respiratory tract	100
Obstructive sleep apnea	55
Decrease in lung capacity	100
Hepatosplenomegaly	100
Stiffness of major and small joints	100
Umbilical or inguinal and inguinal-scrotal hernia	83
Papular eruption on the skin	5
Retinitis pigmentosa	5
Progressive conductive or neurosensory hearing loss	85
Impairments of the nervous system	85
Mental retardation	75
Tonic–clonic convulsions	55

Approximately, 1/5 of the patients had a papular rash with papules filled with glycolipid complexes and localized on the lateral and posterior surfaces of the thighs, shoulders and shoulder blades. This symptom is characteristic of Hunter syndrome which does not occur in patients with other types of mucopolysaccharidosis.

All patients demonstrated high excretion of dermatan sulfate and heparan sulfate in urine and low residual IDS activity in plasma or in dried blood spots.

228 patients from 207 families were completely genotyped. 122 different nucleotide variants were detected: 50 missense, 16 nonsense, 11 splicing substitutions, 27 small deletion, 5 small insertions/duplications, 3 small indels, and 10 gross deletions and complex rearrangements. Some nucleotide variants have been found multiple times in different patients from different families (Table 2). Many disease-associated variants found have been previously described (www.hgmd.cf.ac.uk). From 50 missense substitutions, 12 were not detected before. To evaluate their pathogenic influence, three different programs for impact prediction of nucleotide changes were used. All programs revealed probably deleterious effect of nucleotide variants for all except one change found. For nucleotide variant c.103G>C both programs PolyPhen-2 and PMUT Pathogenic mutation prediction revealed a possibly damaging effect. (Table 3). Additional analysis of nucleotide variant c.103G>C with the program Human Splicing Finder interpreted this variant as the most probably affecting splicing due to Broken WT Donor Site (data not showed). Two different nucleotide variants c.1411G>C and c.1418C>T had been detected in one patient (#43). Both nucleotide variants were novel ones. According to the prediction of pathogenicity, both nucleotide substitutions were probably pathogenic (Table 3). Moreover, five new nonsense substitutions, 19 novel small deletions, 2 novel splicing substitutions, 5 novel small insertion/duplications and 3 novel small indels were found. All small deletions, except the two, all small insertions and one small indel were leading to frame shift and premature stop codon. Two other small indels may have affected splicing. As can be seen from Table 2, the largest number of nucleotide variants was registered in exons 3, 5, 7 and 9. The analysis of nucleotide variants showed that the largest share was represented by point changes (missense or nonsense). Gross rearrangements and major deletions accounted for 14.4%, and 9.6% were splicing substitutions. The nucleotide variants c.253G>A, c.257C>T, c.263G>A, c.263G>T, c.514C>T, c.998C>T, c.1327C>T, c.1402C>T; c.1403G>A and the splicing substitution c.1122C>T were detected more than twice in patients from different families. All these point variants involve CpG sites of the *IDS* gene. The data were consistent with previous studies [19]. The small deletion c.596_599delAACA was detected in five patients from five families. The IDS/IDSP1 inversion has been described in detail [18] and was found in 17 patients from 16 families (see Table 4).

**Table 2:** Nucleotide variants found in the *IDS* gene

Nucleotide change number	Nucleotide; protein change found	Type of nucleotide change	Exons of *IDS* gene	HGMD accession	Allele frequency (%) in presented cohort	Comments
1	c.103G>C; p.Asp35His	Missense	1	None	0.88	Novel (NC_000023.11:g.149505035C>G) ClinVar accession SCV001450592
2	c.136G>T; p.Asp46Tyr	Missense	2	None	0.44	Novel (NC_000023.11:g.149504261C>A) ClinVar accession SCV001450595 one another described in the same codon
3	c.136G>A; p.Asp46Asn	Missense	2	None	0.44	Novel (NC_000023.11:g.149504261C>T) ClinVar accession SCV001450596 one another described in the same codon
4	c.160T>G; p.Tyr54Asp	Missense	2	CM981010	0.44	
5	c.187A>G; p.Asn63Asp	Missense	2	CM960853	1.3	
6	c.236C>A; p.Ala79Glu	Missense	2	CM981012	0.88	
7	c.253G>A; p.Ala85Thr	Missense	3	CM960855	2.2	
8	c.253G>T; p.Ala85Ser	Missense	3	CM981013	0.44	
9	c.257C>T; p.Pro86Leu	Missense	3	CM950659	1.3	
10	c.257C>G; p.Pro86Arg	Missense	3	CM930414	0.44	
11	c.263G>A; p.Arg88His	Missense	3	CM960857	2.65	
12	c.262C>T; p.Arg88Cys	Missense	3	CM950661	1.76	
13	c.263G>T; p.Arg88Leu	Missense	3	CM981014	0.44	
14	c.263G>C; p.Arg88Pro	Missense	3	CM970749	0.44	
15	c.283A>T; p.Arg95Trp	Missense	3	None	0.44	Novel (NC_000023.11:g.149503447T>A) ClinVar accession SCV001450598 three other described in the same codon
16	c.305T>G; p.Leu102Arg	Missense	3	CM981017	0.88	
17	c.307T>G; p.Tyr103Asp	Missense	3	None	0.44	Novel (NC_000023.11:g.149503423A>C) ClinVar accession SCV001450601 two other described in the same codon
18	c.325T>C; p.Trp109Arg	Missense	3	CM128183	0.88	
19	c.359C>G; p.Pro120Arg	Missense	3	CM930417	0.44	
20	c.395C>G; p.Ser132Trp	Missense	3	CM950663	0.88	
21	c.403A>G; p.Lys135Glu	Missense	3	None	0.44	Novel (NC_000023.11:g.149503327T>C) ClinVar accession SCV001450599 two other described in the same codon
22	c.476A>C; p.His159Pro	Missense	4	CM981026	0.44	
23	c.512G>A; p.Cys171Tyr	Missense	5	None	0.44	Novel (NC_000023.11:g.149498303C>T) ClinVar accession SCV001450602 one another described in the same codon
24	c.545T>C; p.Leu182Pro	Missense	5	CM981027	0.44	
25	c.551G>T; p.Cys184Phe	Missense	5	CM960862	0.44	
26	c.587T>C; p.Leu196Ser	Missense	5	CM981029	0.88	
27	c.590C>T; p.Pro197Leu	Missense	5	None	1.76	Novel (NC_000023.11:g.149498225G>A) ClinVar accession SCV001450603
28	c.593A>G; p.Asp198Gly	Missense	5	CM981030	0.44	
29	c.671G>A; p.Gly224Glu	Missense	5	CM981031	0.44	
30	c.697A>G; p.Arg233Gly	Missense	5	CM146285	1.3	
31	c.776T>C; p.Leu259Pro	Missense	6	CM030889	0.44	
32	c.795C>A; p.Asn265Lys	Missense	6	CM128190	0.88	
33	c.795C>G; p.Asn265Lys	Missense	6	CM141180	0.44	
34	c.998C>T; p.Ser333Leu	Missense	7	CM920367	3.09	
35	c.1004A>G; p.His335Arg	Missense	7	CM981045	0.44	
36	c.1006G>C; p.Gly336Arg	Missense	8	CM970753	0.44	
37	c.1019G>A; p.Gly340Asp	Missense	8	CM981048	1.3	
38	c.1028G>A; p.Gly343Glu	Missense	8	None	0.44	Novel (NC_000023.11:g.149487077C>T) ClinVar accession SCV001450616
39	c.1035G>C, p.Trp345Cys	Missense	8	CM950668	0.44	
40	c.1034G>C; p.Trp345Ser	Missense	8	None	0.44	Novel (NC_000023.11:g.149487071C>G) ClinVar accession SCV001450617 four other described in the same codon
41	p.1037C>T; p.Ala346Val	Missense	8	CM950669	1.3	
42	c.1204G>A; p.Glu402Lys	Missense	9	CM167391	0.88	
43	c.1295G>A; p.Cys432Tyr	Missense	9	CM981052	0.88	
44	c.1402C>T; p.Arg468Trp	Missense	9	CM920369	2.2	
45	c.1403G>A; p.Arg468Gln	Missense	9	CM930422	4.4	
46	c.1411G>C; p.Asp471His c.1418C>T; p.Pro473Leu	Missense	9	None	0.44	Novel Novel (NC_000023.11:g.[149482988C>G;149482981G>A]) ClinVar accession SCV001450627
47	c.1432G>T; p.Asp478Tyr	Missense	9	CM981054	0.44	
48	c.1432G>A; p.Asp478Asn	Missense	9	BM1234454	0.44	
49	c.1454T>G; p.Ileu485Arg	Missense	9	CM940967	0.88	
50	c.1565T>C; p.Leu522Pro	Missense	9	HM971766	0.44	
51	c.196C>T; p.Gln66Term	Nonsense	2	CM068304	0.44	
52	c.361C>T; p.Gln121Term	Nonsense	3	CM128174	0.44	
53	c.514C>T; p.Arg172Term	Nonsense	5	CM920366	2.65	
54	c.598C>T p.Gln200Term	Nonsense	6	CM146284	0.88	
55	c.800G>A; p.Trp267Term	Nonsense	6	CM050243	0.44	
56	c.814C>T; p.Gln272Term	Nonsense	6	None	0.44	Novel (NC_000023.11:g.149496411G>A) ClinVar accession SCV001450612
57	c.829C>T; p.Gln277Term	Nonsense	6	None	0.44	Novel (NC_000023.11:g.149496396G>A) ClinVar accession SCV001450613
58	c.998C>A; p.Ser333Term	Nonsense	7	None	0.88	Novel (NC_000023.11:g.149490322G>T) ClinVar accession SCV001450615 two other described in the same codon
59	c.1010G>A; p.Trp337Term	Nonsense	8	CM128194	0.88	
60	c.1234G>T; p.Gly412Term	Nonsense	9	None	0.44	Novel (NC_000023.11:g.149483165C>A) ClinVar accession SCV001450623
61	c.1288G>T; p.Glu430Term	Nonsense	9	CM146287	0.44	
62	c.1327C>T; p.Arg443Term	Nonsense	9	CM920368	1.3	
63	c.1340T>A; p.Leu447Term	Nonsense	9	None	0.44	Novel (NC_000023.11:g.149483059A>T) ClinVar accession SCV001450625
64	c.1375G>T; p.Glu459Term	Nonsense	9	CM1719915	0.44	
65	c.1445T>G; p.Leu482Term	Nonsense	9	CM981058	0.44	
66	c.1608T>A; p.Tyr536Term	Nonsense	9	CM141189	0.44	
67	IVS1 as A-G -2; c.104-2A>G	Splicing substitutions		CS982224	0.44	
68	IVS2 ds G-C +1; c.240+1G>C	Splicing substitutions		CS050391	0.44	
69	IVS2 ds G-T +1; c.240+1G>T	Splicing substitutions		CS982227	0.44	
70	IVS2 as C-G -9; c.241-9C>G	Splicing substitutions		None	0.88	Novel (NC_000023.11:g.149503498G>C) ClinVar accession SCV001450634
71	IVS4 ds G-A +1; c.507+1G>A	Splicing substitutions		CS982228	0.88	
72	IVS6 ds G-A +1; c.879+1G>A	Splicing substitutions		CS982229	0.44	
73	IVS6 as A-G -2; c.880-2A>G	Splicing substitutions		CS930833	0.44	
74	IVS7 ds T-G +2 c.1006+2T>G	Splicing substitutions		None	0.44	Novel (NC_000023.11:g.149490312A>C) ClinVar accession SCV001450635
75	IVS7 as G-A -1; c.1007-1G>A	Splicing substitutions		CS120471	0.44	
76	IVS8 as C-A -15; c.1181-15C>A	Splicing substitutions		CS013824	0.44	
77	IVS8 ds C-T -59; c.1122C>T	Splicing substitutions		CS963080	5.75	
78	c.118_120delCTT; p.Leu40del	Small deletion	2	None	0.44	Novel (NC_000023.11:g.149504277_149504279delAAG) ClinVar accession SCV001450593
79	c.121_123delCTC p.Leu41del	Small deletion	2	CD941707	0.44	
80	c.133delG; p.Asp45Metfs	Small deletion	2	None	0.44	Novel (NC_000023.11:g.149504264delC) ClinVar accession SCV001450594
81	c.248delT; p.Val83Glyfs	Small deletion	3	None	0.44	Novel (NC_000023.11:g.149503482delA) ClinVar accession SCV001450597
82	c.305delT; p.Leu102Argfs	Small deletion	3	None	0.44	Novel (NC_000023.11:g.149503425delA) ClinVar accession SCV001450600
83	c.404_405delAA; p.Lys135Serfs	Small deletion	3	None	0.44	Described ClinVar accession VCV000499561.1
84	c.410_411delTT p.Phe137Serfs	Small deletion	3	CD012530	0.44	
85	c.596_599delAACA; p.Lys199Argfs	Small deletion	5	CD941708	2.21	
86	c.613delG; p.Ala205Profs	Small deletion	5	None	0.44	Novel (NC_000023.11:g.149498202delC) ClinVar accession SCV001450604
87	c.625_627del TTG; p.Leu209del	Small deletion	5	None	0.44	Novel (NC_000023.11:g.149498188_149498190delCAA) ClinVar accession SCV001450605
88	c.667_683del17; p.Val223Thrfs	Small deletion	5	CD982702	0.44	
89	c.687delC; p.His229Glnfs	Small deletion	5	None	0.44	Novel (NC_000023.11:g.149498128delG) ClinVar accession SCV001450606
90	c.715_721del7; p.Gln239Cysfs	Small deletion	6	None	0.44	Novel (NC_000023.11:g.149496504_149496510delACTTCTG) ClinVar accession SCV001450607
91	c.782delC; p.Pro261Leufs	Small deletion	6	CD982703	0.44	
92	c.800_801delGG; p.Trp267Tyrfs	Small deletion	6	None	0.44	Novel (NC_000023.11:g.149496424_149496425delCC) ClinVar accession SCV001450611
93	c.899_900delAC; p.Tyr300Phefs	Small deletion	7	None	0.44	Novel (NC_000023.11:g.149490420_149490421delGT) ClinVar accession SCV001450614
94	c.908_909delCT; p.Ser303Cysfs	Small deletion	7	CD1412401	0.44	
95	c.1077delG; p.Ile360Tyrfs	Small deletion	8	CD146296	0.44	
96	c.1129delC; p.Leu377Phefs	Small deletion	8	None	0.44	Novel (NC_000023.11:g.149486976delG) ClinVar accession SCV001450618
97	c.1191delC; p.Met398Trpfs	Small deletion	9	None	0.44	Novel (NC_000023.11:g.149483208delG) ClinVar accession SCV001450620
98	c.1214_1220del7; p.Ser405Phefs	Small deletion	9	None	0.44	Novel (NC_000023.11:g.149483179_149483185delAAAAGAG) ClinVar accession SCV001450621
99	c.1221delT; p.Ser409Argfs	Small deletion	9	None	0.44	Novel (NC_000023.11:g.149483178delA) ClinVar accession SCV001450622
100	c.1353_1357delGTACC; p.Tyr452Profs	Small deletion	9	None	0.44	Novel (NC_000023.11:g. 149483042_149483046delGGTAC) ClinVar accession SCV001450626
101	c.1426_1437 del12 p.476_479delAsnSerAspLys	Small deletion	9	None	0.44	Novel (NC_000023.11:g.149482962_149482973delCTTGTCAGAATT) ClinVar accession SCV001450629
102	c.1431delT; p.Asp478Thrfs	Small deletion	9	None	0.44	Novel (NC_000023.11:g.149482968delA) ClinVar accession SCV001450628
103	c.1438_1442delCCGAG;p.Pro480Phefs	Small deletion	9	None	0.44	Novel (NC_000023.11:g.149482957_149482961delCTCGG) ClinVar accession SCV001450630
104	c.1466delG p.Val489Alafs	Small deletion	9	CD146297	0.44	
105	c.776_777dupTA; p.Pro260Tyrfs	Small insertions/duplications	6	None	0.44	Novel (NC_000023.11:g.149496448_149496449dupTA) ClinVar accession SCV001450608
106	c.801_802insG; p.Met268Aspfs	Small insertions/duplications	6	None	0.44	Novel (NC_000023.11:g.149496423_149496424insC) ClinVar accession SCV001450610
107	c.1151_1152 insTGCGACCCTTT; p.Phe384Leufs	Small insertions/duplications	8	None	0.44	Novel (NC_000023.11:g.149486954_149486955 insTGCGACCCTTT) ClinVar accession SCV001450619
108	c.1239_c.1240insCT; p.Ala414Leufs	Small insertions/duplications	9	None	0.44	Novel (NC_000023.11:g.149483159_149483160insAG) ClinVar accession SCV001450624
109	c.1491_1492dupTA; p.Arg498Ileufs	Small insertions/duplications	9	None	0.44	Novel (NC_000023.11:g.149482909_149482910dupTA) ClinVar accession SCV001450631
110	c.104-1_104delGAinsT	Small indels	2	None	0.44	Novel (NC_000023.11:g.149504293_149504294delTCinsA) ClinVar accession SCV001450632
111	c.240 +2_c.240+3insTCCCAGA	Small indels	Intron 2	None	0.44	Novel (NC_000023.11:g.149504154_149504155insTCCCAGA) ClinVar accession SCV001450633
112	c.786_787delGGinsC; p.Ala263Profs	Small indels	6	None	0.44	Novel (NC_000023.11:g.149496438_149496439delCCinsG) ClinVar accession SCV001450609
113	gDNA level exons 1–3 deletion	Gross deletions			0.88	
114	gDNA level exons 1–4 deletion	Gross deletions			0.44	
115	gDNA level exons 1–7 deletion	Gross deletions			0.44	
116	gDNA level exons 1–7 deletion	Gross deletions			0.44	
117	gDNA level exon 4 deletion	Gross deletions			0.44	
118	cDNA level del incl. ex 5–6	Gross deletions		CG984375	0.44	
119	gDNA level exon 7 deletion	Gross deletions			0.44	
120	Complete IDS del	Gross deletions			3.5	
121	Recomb. between in. 7 and seq. distal of ex. 3 in IDS-2 without exons deletion	Complex rearrangements		CP973598	6.19	
122	Recomb. between in. 7 and seq. distal of ex. 3 in IDS-2 with 4–7 exons deletion	Complex rearrangements			1.32

**Table 3:** Prediction of functional effects of 12 missense substitutions found

Nucleotide changes number	Nucleotide/protein change	Programs for prediction of nucleotide changes		
		PolyPhen-2	PMUT pathogenic mutation prediction	Mutation tester
1	c.103G>C; p.Asp35His	Possibly damaging with a score of 0.710	0.47 (83%) Neutral	Disease causing
2	c.136G>T; p.Asp46Tyr	Probably damaging with a score of 1.000	0.94 (94%) Disease	Disease causing
3	c.136G>A; p.Asp46Asn	Probably damaging with a score of 1.000	0.89 (92%) Disease	Disease causing
4	c.283A>T; p.Arg95Trp	Probably damaging with a score of 1.000	0.79 (88%) Disease	Disease causing
5	c.307T>G; Tyr103Asp	Probably damaging with a score of 1.000	0.82 (90%) Disease	Disease causing
6	c.403A>G; p.Lys135Glu	Probably damaging with a score of 1.000	0.94 (94%) Disease	Disease causing
7	c.512G>A; p.Cys171Tyr	Probably damaging with a score of 1.000	0.53 (80%) Disease	Disease causing
8	c.590C>T; p.Pro197Leu	Probably damaging with a score of 0.996	0.69 (86%) Disease	Disease causing
9	c.1028G>A; p.Gly343Glu	Probably damaging with a score of 0.996	0.83 (90%) Disease	Disease causing
10	c.1034G>C; p.Trp345Ser	Probably damaging with a score of 1.000	0.89 (92%) Disease	Disease causing
11	c.1411G>C; p.Asp471His	Probably damaging with a score of 0.999	0.81 (89%) Disease	Disease causing
12	c.1418C>T; p.Pro473Leu	Probably damaging with a score of 0.976	0.72 (86%) Disease	Disease causing

**Table 4:** Mutation spectrum in the *IDS* gene in patients with MPS II

Family number	Patient number	Age at diagnosis	Enzyme activity in plasma (N = 297–705 nmol/4h/ml)	Enzyme activity in dried blood spots (N = 2.5–50 μmM/l/h)	Phenotype	Nucleotide/protein change in *IDS* gene
1	1	7	16.2		Mild	c.187A>G; p.Aspn63Asp
2	2	31	21.1		Mild	c.187A>G; p.Aspn63Asp
	3	32	18.2		Mild	c.187A>G; p.Aspn63Asp
3	4	8	1.2		Mild	c.236C>A; p.Ala79Glu
	5	10	8.4		Mild	c.236C>A; p.Ala79Glu
4	6	11	7.4		Mild	c.305T>G; p.Leu102Arg
	7	9	8.1		Mild	c.305T>G; p.Leu102Arg
5	8	55	–	0.01	Mild	c.590C>T; p.Pro197Leu
	9	12	–	0.01	Mild	c.590C>T; p.Pro197Leu
	10	5	–	0.01	Mild	c.590C>T; p.Pro197Leu
	11	11	–	0.01	Mild	c.590C>T; p.Pro197Leu
6	12	16	14.7		Mild	c.593A>G; p.Asp198Gly
7	13	23	8.4		Mild	c.1019G>A; p.Gly340Asp
	14	19	6.1		Mild	c.1019G>A; p.Gly340Asp
8	15	33	11.7		Mild	c.1019G>A; p.Gly340Asp
9	16	35	0.01		Mild	p.1037C>T; p.Ala346Val
	17	23	0.05		Mild	p.1037C>T; p.Ala346Val
	18	18	0.07		Mild	p.1037C>T; p.Ala346Val
10	19	15	3.6		Mild	c.1234G>T; p.Gly412Term
11	20	11	9.4		Mild -> intermediate	c.253G>A; p.Ala85Thr
12	21	9	10.4		Mild -> intermediate	c.253G>A; p.Ala85Thr
13	22	6	18.6		Mild -> intermediate	c.253G>A; p.Ala85Thr
14	23	9	14.4		Mild -> intermediate	c.253G>A; p.Ala85Thr
15	24	13	0.01		Mild -> intermediate	c.253G>A; p.Ala85Thr
16	25	8	0.02		Mild -> intermediate	c.253G>T; p.Ala85Ser
17	26	13	11.3		Mild -> intermediate	c.587T>C; p.Leu196Ser
18	27	5	4.7		Mild -> intermediate	c.587T>C; p.Leu196Ser
19	28	2	2.1		Mild -> intermediate	c.1204G>A; p.Glu402Lys
20	29	3	1.4		Mild -> intermediate	c.1204G>A; p.Glu402Lys
21	30	4	–	0.01	Mild -> intermediate	c.1438_1442delCCGAG; p.Pro480Phefs
22	31	28	14.8		Mild -> intermediate	c.1028G>A; p.Gly343Glu
23	32	11	7.4		Mild -> intermediate	c.1034G>C; p.Trp345Ser
24	33	25	4.8		Mild -> intermediate	c.1035G>C, p.Trp345Cys
25	34	8	5.1		Mild -> intermediate	IVS2 as C-G -9; c.241-9C>G
	35	10	5.2		Mild -> intermediate	IVS2 as C-G -9; c.241-9C>G
26	36	7	0.1		Intermediate -> severe	c.283A>T; p.Arg95Trp
27	37	7	0.01		Intermediate -> severe	c.512G>A; p.Cys171Tyr
28	38	5	8.4		Intermediate -> severe	c.545T>C; p.Leu182Pro
29	39	6	2.3		Intermediate -> severe	c.551G>T; p.Cys184Phe
30	40	6	2.7		Intermediate -> severe	c.1327C>T; p.Arg443Term
31	41	7	3.18		Intermediate -> severe	c.1327C>T; p.Arg443Term
32	42	2	–	0.01	Intermediate -> severe	c.1327C>T; p.Arg443Term
33	43	13	21.6		Intermediate -> severe	c.1411G>C; p.Asp471His c.1418C>T; p.Pro473Leu
34	44	14	2.2		Intermediate -> severe	IVS6 ds G-A +1; c.879+1G>A
35	45	11	0.01		Intermediate -> severe	IVS8 ds C-T -59; c.1122C>T
36	46	11	0.01		Intermediate -> severe	IVS8 ds C-T -59; c.1122C>T
37	47	6	0.02		Intermediate -> severe	IVS8 ds C-T -59; c.1122C>T
38	48	8	0.01		Intermediate -> severe	IVS8 ds C-T -59; c.1122C>T
39	49	4	0.01		Intermediate -> severe	IVS8 ds C-T -59; c.1122C>T
40	50	5	2.2		Intermediate -> severe	IVS8 ds C-T -59; c.1122C>T
41	51	3	0.01		Intermediate -> severe	IVS8 ds C-T -59; c.1122C>T
42	52	7	0.01		Intermediate -> severe	IVS8 ds C-T -59; c.1122C>T
43	53	18	0.01		Intermediate -> severe	IVS8 ds C-T -59; c.1122C>T
44	54	4	4.9		Intermediate -> severe	IVS8 ds C-T -59; c.1122C>T
45	55	7	2.3		Intermediate -> severe	IVS8 ds C-T -59; c.1122C>T
46	56	4	7.2		Intermediate -> severe	IVS8 ds C-T -59; c.1122C>T
47	57	5			Intermediate -> severe	IVS8 ds C-T -59; c.1122C>T
48	58	3	4.9		Intermediate -> severe	c.118_120delCTC; p.Leu40del
49	59	4	21.4		Intermediate -> severe	c.121_123delCTC p.Leu41del
50	60	6	0.01		Intermediate -> severe	c.625_627del TTG p.Leu209del
51	61	9	4.4		Intermediate -> severe	c.1426_1437 del12; p.476_479delAsnSerAspLys
52	62	6	8.3		Severe	c.103G>C; p.Asp35His
	63	4	10.4		Severe	c.103G>C; p.Asp35His
53	64	3	0.35		Severe	c.136G>T; p.Asp46Tyr
54	65	3	–	0.01	Severe	c.136G>A; p.Asp46Asn
55	66	4	0.1		Severe	c.160T>G; p.Tyr54Asp
56	67	4	4.6		Severe	c.257C>T; p. Pro86Leu
57	68	5	5.1		Severe	c.257C>T; p. Pro86Leu
58	69	2	3.5		Severe	c.257C>T; p. Pro86Leu
59	70	10	11.8		Severe	c.257C>G; p. Pro86Arg
60	71	6	0.01		Severe	c.263G>A; p.Arg88His
	72	3	0.01		Severe	c.263G>A; p.Arg88His
61	73	3	0.7		Severe	c.263G>A; p.Arg88His
	74	4	0.8		Severe	c.263G>A; p.Arg88His
62	75	7	–	0.01	Severe	c.263G>A; p.Arg88His
63	76	3	0.01		Severe	c.263G>A; p.Arg88His
64	77	5	0.01		Severe	c.263G>T; p.Arg88Leu
65	78	4	0.02		Severe	c.262C>T; p.Arg88Cys
66	79	5	0.01		Severe	c.262C>T; p.Arg88Cys
67	80	4	0.01		Severe	c.262C>T; p.Arg88Cys
68	81	3	0.01		Severe	c.262C>T; p.Arg88Cys
69	82	4	3.2		Severe	c.263G>C; p.Arg88Pro
70	83	8	0.01		Severe	c.307T>G; p.Tyr103Asp
71	84	5	4.8		Severe	c.325T>C; p.Trp109Arg
72	85	6	1.37		Severe	c.325T>C; p.Trp109Arg
73	86	1	–	0.01	Severe	c.359C>G; p.Pro120Arg
74	87	4	0.01		Severe	c.395C>G; p.Ser132Trp
75	88	9	0.01		Severe	c.395C>G; p.Ser132Trp
76	89	3	0.01		Severe	c.403A>G; p.Lys135Glu
77	90	10	0.1		Severe	c.476A>C; p.His159Pro
78	91	5	0.01		Severe	c.671G>A; p.Gly224Glu
79	92	5	1.2		Severe	c.697A>G; p.Arg233Gly
	93	2	1.59		Severe	c.697A>G; p.Arg233Gly
80	94	1.5	0.01		Severe	c.697A>G; p.Arg233Gly
81	95	15	4.1		Severe	c.776T>C; p.Leu259Pro
82	96	3	0.15		Severe	c.795C>A; p.Asn265Lys
83	97	3	0.49		Severe	c.795C>A; p.Asn265Lys
84	98	3	10.8		Severe	c.795C>G; p.Asn265Lys
85	99	10	0.01		Severe	c.998C>T; p.Ser333Leu
86	100	7	0.01		Severe	c.998C>T; p.Ser333Leu
87	101	6	0.01		Severe	c.998C>T; p.Ser333Leu
88	102	7	0.01		Severe	c.998C>T; p.Ser333Leu
89	103	5	0.01		Severe	c.998C>T; p.Ser333Leu
90	104	7	0.01		Severe	c.998C>T; p.Ser333Leu
91	105	1	3.5		Severe	c.998C>T; p.Ser333Leu
92	106	2	0.01		Severe	c.1004A>G; p.His335Arg
93	107	5	2.7		Severe	c.1006G>C; p.Gly336Arg
94	108	10	0.1		Severe	c.1295G>A; p.Cys432Tyr
95	109	4	0.01		Severe	c.1295G>A; p.Cys432Tyr
96	110	4	5.4		Severe	c.1402C>T; p.Arg468Trp
97	111	2	0.1		Severe	c.1402C>T; p.Arg468Trp
98	112	3	0.01		Severe	c.1402C>T; p.Arg468Trp
99	113	5	0.1		Severe	c.1402C>T; p.Arg468Trp
100	114	3	–	0.01	Severe	c.1402C>T; p.Arg468Trp
101	115	3	0.01		Severe	c.1403G>A; p.Arg468Gln
102	116	3	0.9		Severe	c.1403G>A; p.Arg468Gln
	117	3	0.01		Severe	c.1403G>A; p.Arg468Gln
103	118	5	1.4		Severe	c.1403G>A; p.Arg468Gln
104	119	2	0.01		Severe	c.1403G>A; p.Arg468Gln
105	120	2	6.3		Severe	c.1403G>A; p.Arg468Gln
	121	4	2.8		Severe	c.1403G>A; p.Arg468Gln
106	122	3	10.8		Severe	c.1403G>A; p.Arg468Gln
107	123	4	0.01		Severe	c.1403G>A; p.Arg468Gln
108	124	2	3.12		Severe	c.1403G>A; p.Arg468Gln
109	125	4	0.01		Severe	c.1432G>T; p.Asp478Tyr
110	126	7	0.1		Severe	c.1432G>A; p.Asp478Asn
111	127	6	0.01		Severe	c.1454T>G; p.Ileu485Arg
112	128	6	0.01		Severe	c.1454T>G; p.Ileu485Arg
113	129	8	0.01		Severe	c.1565T>C; p.Leu522Pro
114	130	3	0.15		Severe	c.196C>T; p.Gln66Term
115	131	2	0.01		Severe	c.361C>T; p.Gln121Term
116	132	3	4.5		Severe	c.514C>T; p.Arg172Term
117	133	4	2.7		Severe	c.514C>T; p.Arg172Term
118	134	2	7.8		Severe	c.514C>T; p.Arg172Term
119	135	4	4.32		Severe	c.514C>T; p.Arg172Term
120	136	4	8.4		Severe	c.514C>T; p.Arg172Term
121	137	2	–	0.01	Severe	c.514C>T; p.Arg172Term
122	138	6	0.01		Severe	c.598C>T p.Gln200Term
123	139	2	2.1		Severe	c.598C>T p.Gln200Term
124	140	12	0.9		Severe	c.800G>A; p.Trp267Term
125	141	5	0.01		Severe	c.814C>T; p.Gln272Term
126	142	4	2.1		Severe	c.829C>T; Gln277Term
127	143	8	18		Severe	c.998C>A; p.Ser333Term
	144	2	10.1		Severe	c.998C>A; p.Ser333Term
128	145	3	0.18		Severe	c.1010G>A; p.Trp337Term
129	146	3	4.4		Severe	c.1010G>A; p.Trp337Term
130	147	1	0.07		Severe	c.1288G>T; p.Glu430Term
131	148	6	0.01		Severe	c.1340T>A; p.Leu447Term
132	149	3	0.01		Severe	c.1375G>T; p.Glu459Term
133	150	3.5	0.01		Severe	c.1445T>G; p.Leu482Term
134	151	6	0.01		Severe	c.1608T>A;p.Tyr536Term
135	152	4	0.01		Severe	IVS1 as A-G -2; c.104-2A>G
136	153	4	0.01		Severe	IVS2 ds G-C +1; c.240+1G>C
137	154	4	4.8		Severe	IVS2 ds G-T +1; c.240+1G>T
138	155	7	0.01		Severe	IVS4 ds G-A +1; c.507+1G>A
139	156	16	3.96		Severe	IVS4 ds G-A +1; c.507+1G>A
140	157	9	3.6		Severe	IVS6 as A-G -2; c.880-2A>G
141	158	4	1.14		Severe	IVS7 ds T-G +2c.1006+2T>G
142	159	5	0.01		Severe	IVS7 as G-A -1; c.1007-1G>A
143	160	5	0.01		Severe	IVS8 as C-A -15; c.1181-15C>A
144	162	6	2.1		Severe	c.133delG p.Asp45Metfs
145	163	1.5	0.12		Severe	c.248delT; p.Val83Glyfs
146	164	3	0.01		Severe	c.305delT; p.Leu102Argfs
147	165	2	1.4		Severe	c.404_405delAA; p.Lys135Serfs
148	166	2	2.52		Severe	c.410_411delTT p.Phe137Sfs
149	167	6	4.2		Severe	c.596_599delAACA;p.(Lys199Argfs)
150	168	9	0.58		Severe	c.596_599delAACA;p.(Lys199Argfs)
151	169	7	1.2		Severe	c.596_599delAACA;p.(Lys199Argfs)
152	170	5	9.1		Severe	c.596_599delAACA;p.(Lys199Argfs)
153	171	4	0.7		Severe	c.596_599delAACA;p.(Lys199Argfs)
154	172	3	18.1		Severe	c.613delG; p.Ala205Profs
155	173	9	0.01		Severe	c.667-683del17; p.Val223Thrfs
156	174	5	0.01		Severe	c.687delC; p.His229Glnfs
157	175	6	2.3		Severe	c.715_721del7; p.Gln239Cysfs
158	176	2	0.01		Severe	c.782delC p.Pro261Lfs
159	177	13	0.66		Severe	c.800_801delGG; p.Trp267Tyrfs
160	178	4	0.01		Severe	c.899_900delAC; p.Tyr300Phefs
161	179	8	8.04		Severe	c.908_909delCT; p.Ser303Cysfs
162	180	1	0.43		Severe	c.1077delG; p.Ile360Tyrfs
163	181	12	12.6		Severe	c.1129delC p.Leu377Phefs
164	182	4	0.01		Severe	c.1191delC p.Met398Trpfs
165	183	6	0.01		Severe	c.1214_1220del7 p.Leu406Hisfs
166	184	3	2.3		Severe	c.1221delT p.Cys409Arg fs
167	185	16	0.79		Severe	c.1353_1357delGTACCp.Tyr452Profs
168	186	3	2.4		Severe	c.1431delT; p.Asp478Thrfs
169	187	3	1.9		Severe	c.1466delG p.Val489Alafs
170	188	3	–	0.01	Severe	c.776_777dupTA; p.Pro260Tyrfs
171	189	5	0.01		Severe	c.801_802insG; p.Met268Aspfs
172	190	4	1.3		Severe	c.1151_1152 ins11; p.Phe384Leufs
173	191	9	6.6		Severe	c.1239_1240insCT; p.Ala414Leufs
174	192	5	0.01		Severe	c.1491_1492dupTA; p.Arg498Ileufs
175	193	4	2.64		Severe	del c.104-1_104delGAinsT
176	194	8	0.01		Severe	c.240+2_240+3 insTCCCAGA (intron 2)
177	195	4	0.48		Severe	c.786-787delGGinsC; p.Ala263Profs
178	196	2	–	0.01	Severe	gDNA level exons 1–3 deletion
179	197	3	–	0.01	Severe	gDNA level exons 1–3 deletion
180	198	7	1.9		Severe	gDNA level exons 1–4 deletion
181	199	4	0.01		Severe	gDNA level exons 1–7 deletion
	200	1	0.01		Severe	gDNA level exons 1–7 deletion
182	201	2	4.2		Severe	gDNA level exon 4 deletion
183	202	5	0.01		Severe	cDNA level del incl. ex 5–6
184	203	2	9.6		Severe	gDNA level exon 7 deletion
185	204	5	0.01		Severe	Complete IDS del
	205	3	0.01		Severe	Complete IDS del
186	206	2	1.3		Severe	Complete IDS del
187	207	3	0.01		Severe	Complete IDS del
188	208	7	0.01		Severe	Complete IDS del
189	209	10	0.01		Severe	Complete IDS del
190	210	3	0.01		Severe	Complete IDS del
191	211	2	0.01		Severe	Complete IDS del
192	212	5	0.01		Severe	Recomb. between in. 7 and seq. distal of ex. 3 in IDS-2 without exons deletion
193	213	2	0.01		Severe	Recomb. between in. 7 and seq. distal of ex. 3 in IDS without exons deletion
194	214	3	0.01		Severe	Recomb. between in. 7 and seq. distal of ex. 3 in IDS without exons deletion
195	215	6	2.5		Severe	Recomb. between in. 7 and seq. distal of ex. 3 in IDS without exons deletion
196	216	6	7.2		Severe	Recomb. between in. 7 and seq. distal of ex. 3 in IDS without exons deletion
197	217	6	0.43		Severe	Recomb. between in. 7 and seq. distal of ex. 3 in IDS without exons deletion
198	218	3	2.4		Severe	Recomb. between in. 7 and seq. distal of ex. 3 in IDS without exons deletion
199	219	3	0.01		Severe	Recomb. between in. 7 and seq. distal of ex. 3 in IDS without exons deletion
	220	13	3.6		Severe	Recomb. between in. 7 and seq. distal of ex. 3 in IDS without exons deletion
200	221	5	0.01		Severe	Recomb. between in. 7 and seq. distal of ex. 3 in IDS without exons deletion
201	222	2	0.01		Severe	Recomb. between in. 7 and seq. distal of ex. 3 in IDS without exons deletion
202	223	1	5.1		Severe	Recomb. between in. 7 and seq. distal of ex. 3 in IDS without exons deletion
203	224	5	2.3		Severe	Recomb. between in. 7 and seq. distal of ex. 3 in IDS without exons deletion
204	225	1	0.01		Severe	Recomb. between in. 7 and seq. distal of ex. 3 in IDS without exons deletion
205	226	3	0.01		Severe	Recomb. between in. 7 and seq. distal of ex. 3 in IDS with 3–7 exons deletion
206	227	2	0.01		Severe	Recomb. between in. 7 and seq. distal of ex. 3 in IDS with 3–7 exons deletion
207	228	3	0.12		Severe	Recomb. between in. 7 and seq. distal of ex. 3 in IDS with 3–7 exons deletion

The comparative analysis of the severity of clinical symptoms with the results of nucleotide variants detected in the *IDS* gene are illustrated in Table 4. It demonstrates that most of nucleotide variants found caused the development of severe forms of the disease characterized by early (from the first months of life) manifestation of the disease, severe damage to vital organs and body systems, severely reduced intelligence and shorter life expectancy.

The protein changes p.Aspn63Asp (three patients; two families), p.Ala79Glu, (two patients; one family), p.Ala85Thr, p.Leu102Arg (two patients; one family), p.Asp198Gly, p.Gly412Term, p.Pro197Leu (four patients; one family), p.Gly340Asp, p.Ala346Val (three patients; one family), p.Arg443Term, the small deletion without frame shift and site-splicing substitution IVS2-9 c->g led to the development of less severe cases with later symptom manifestations, milder symptoms and higher IQ scores. Our data are consistent with the studies of other researchers who analyzed the influence of amino acid substitution on the *IDS* structure [20, 21].

A brief summary of some patients with a mild form of Hunter syndrome is presented in Table 5.

**Table 5:** Summary of some patients with a mild form and a mild -> intermediate form of Hunter syndrome

Family #	Patient #	Age at diagnosis	Enzyme activity in plasma (N = 297–705 nmol/4h/ml)	Nucleotide variant in *IDS* gene	Education	Profession	Date of death	Comment
3	4	10	1.2	c.236C>A (p.Ala79Glu)	University	Teacher	43	–
	5	8	8.4	c.236C>A (p.Ala79Glu)	University	Economist (38 years old)	–	Active life
4	6	11	7.4	c.305T>G (p.Leu102Pro)	University	Economist 35 years old)	–	Active life
	7	9	8.1	c.305T>G (p.Leu102Pro)	University	Economist (33 years old)	–	Active life
10	19	15	3.6	c.1234G>T (p.Gly412Term)	University	Lawyer (32 years old)	–	Active life
20	29	3	1.4	c.1204G>A (p.Glu402Lys)	University	Lawyer	23	The cause of death is unprofessional tracheal intubation, carried out with the aim of removing the patient from the epileptic status
15	24	13	0.01	c.253G>A (p.Ala85Thr)	historic	Historian	29	Sudden death from acute cardiovascular failure

### Clinical case

**Fig. 1:**
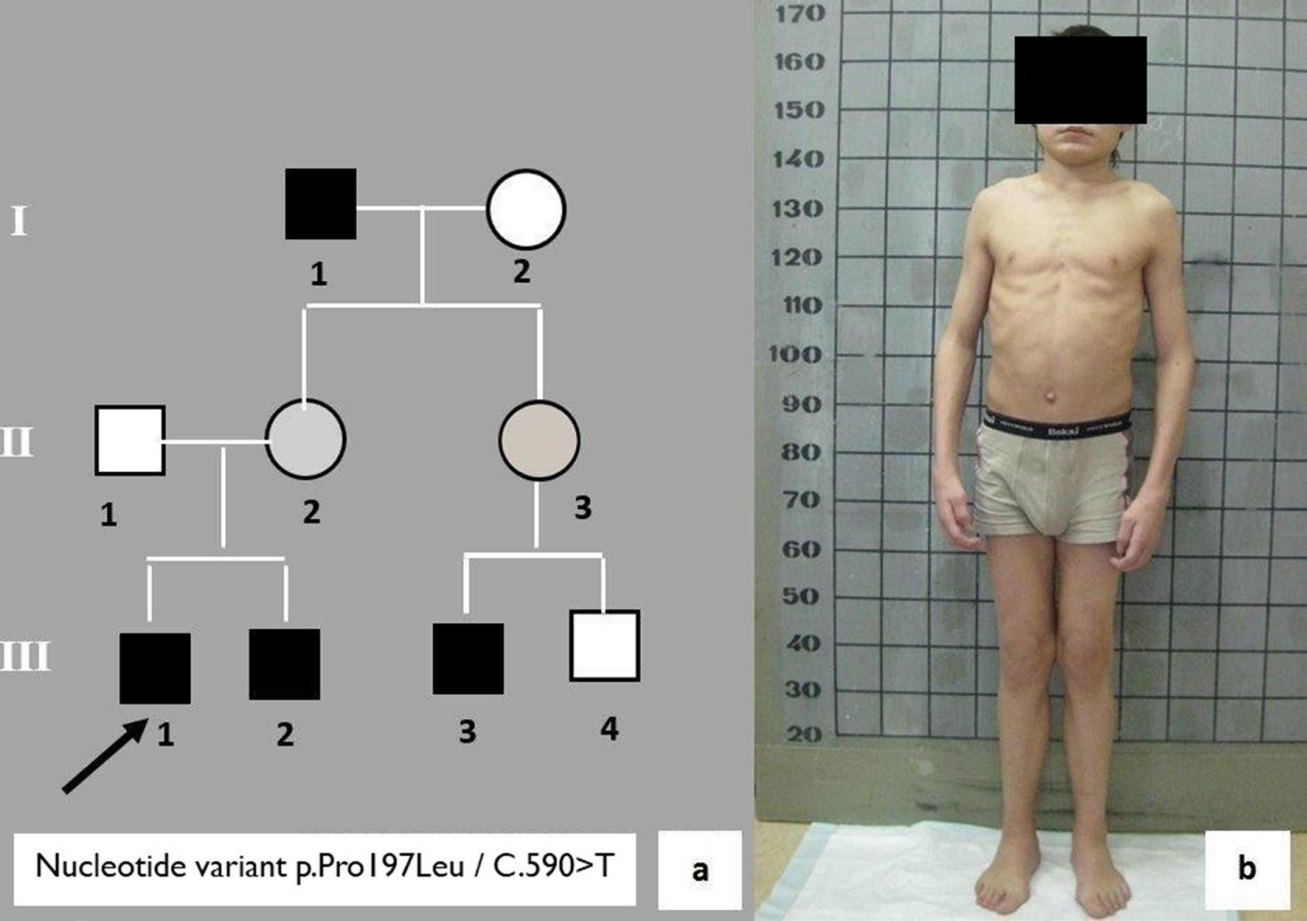
**a** A fragment of the pedigree of patient #16 with a mild form of Hunter syndrome; **b** a 12-year old patient #16 with a mild form of Hunter syndrome

A 12-year old male (#9) was admitted to the genetics department with complaints about rough facial features and stiffness of major and small joints (Fig. 1). While collecting the genealogical history, it was found that the proband’s grandfather on the maternal line exhibits identical symptoms (Fig. 1a—I, 1). The grandfather was 55 years old at the time. The man had a disability and was observed by physicians at the place of residence. The diagnoses he was given were rheumatoid arthritis, hypothyroidism, osteochondrosis, and hypochondroplasia. The proband’s 3-year-old younger brother (Fig. 1a—III, 2) and 4- and 2-year old male cousins (Fig. 1a—III, 3 and III, 4) were considered healthy. Based on the genealogical history and clinical features, the proband was suspected to have mucopolysaccharidosis type II. Examination revealed high rates of renal excretion of heparansulfate and dermatansulfate, a decrease in the activity of I2S in dried blood spots (0.1 μmM/l/h, with the norm being 2.5–50 μmM/l/h) and a missense variant c.590C>T (p.Pro197Leu) in exon 5 of the *IDS* gene was found; so the diagnosis was confirmed. This nucleotide variant was not described before. The examination of men in this family allowed us to diagnose Hunter syndrome in the grandfather (I, 1), the sibling (III, 2) and the cousin (III, 3) of the proband. Ten years of observation of affected members of this family showed a good social adaptation of patients and long life expectancy of the grandfather. The proband successfully completed a technical college. His sibling and cousin are community college students. All affected family members voluntarily refused to receive enzyme replacement therapy. The clinical observation of the family continues.

The rare case of Hunter syndrome in girls observed by the authors is due to a disease-associated variant in the *IDS* gene inherited from the mother and a deletion in the long arm of the X chromosome of paternal origin. The diagnosis was confirmed based on the detection of the absence of enzyme activity of iduronate-2-sulfatase and results of cytogenetic, molecular cytogenetic and molecular genetic examination [22].

## Discussion

We present the results of the clinical observation of 228 Russian patients with Hunter syndrome. The diagnosis of mucopolysaccharidosis type II (Hunter syndrome) consisted of four consecutive stages: 1—Assessment of phenotypic characters; 2—Determination of indicators of excretion of urinary glycosaminoglycans and their fractions, primarily heparan and dermatan sulfates; 3—Measurement of the activity of the lysosomal enzyme iduronate-2-sulfatase; 4—DNA diagnostics, including the search for nucleotide substitutions in the *IDS* gene with an estimate of its frequency and pathogenicity according to the International HMGD Database.

According to the latest concept that MPS II is a continuum between the mild and severe form of the disease, we divided all patients into four groups. Group 1 includes 166 patients with a severe form of the disease, Group 2—patients with a moderate form of the disease, which was subdivided into Group 2a—26 patients with an intermediate -> severe (rather severe) and Group 2b—16 patients with a mild -> intermediate (rather a mild form). Group 3 includes 19 patients with a mild form of Hunter syndrome.

The clinical symptoms of patients with a severe form of mucopolysaccharidosis type II were characterized by an early manifestation of the disease (first months of life), rapid progression of clinical symptoms with the severe damage to the musculoskeletal system, cardiovascular and bronchopulmonary systems, parenchymal organs, hearing, and the formation of rapidly progressing umbilical, inguinal and inguinal-scrotal hernias requiring urgent surgical intervention. These patients completely lacked the ability to move independently and communicate with others; their IQ did not exceed 10–15 points, and life expectancy was short.

The residual activity of I2S in plasma varied from undetectable values to 11.8 nmol/4h/ml. No residual activity in dried blood spots was determined.

Complete deletions of the *IDS* gene, deletions of several exons, site splicing disease-associated variants, nonsense and missense nucleotide substitutions have been detected in patients with a severe form of the disease (Table 4). Many missense nucleotide variants found in patients with a severe form of the disease have been already described (Table 2). The data of other researchers regarding genotype–phenotype correlations are consistent with our findings [20, 21, 23–27]. Five undescribed missense variants were found in patients with a severe form (c.103G>C; c.136G>T; c.136G>A; c.307T>G; c.403A>G). Some nucleotide substitutions were in the same codon where other changes were found. According to the software analysis for impact prediction of nucleotide changes c.136G>T; c.136G>A; c.307T>G; c.403A>G were probably damaging. For the nucleotide change c.103G>C, PolyPhen-2 and PMUT Pathogenic mutation prediction programs showed a possibly damaging effect. However, this nucleotide variant was predicted as most probably affecting splicing by the Human Splicing Finder software. This seems to us to be a more truthful interpretation since patients had a severe form of the disease. Of course, for complete understanding of the damaging effect of nucleotide variants found additional molecular genetic studies are required. Four novel nonsense variants: c.814C>T; c.829C>T; c.998C>A; c.1340T>A and one new site splicing substitution c.1006+2T>G have been found in patients with a severe form of the disease. All previously undescribed small deletions, small insertions and small indels found in the patients with a severe form resulted in the frame shift and premature stop codon, which is expected to lead to the development of a severe form of the disease. It was established that as a result of extended deletions, the synthesis of the truncated protein occurs, which leads to the violation of its full function, and, thus, to the formation of severe clinical symptoms.

The 2a group of patients with an intermediate -> severe (rather severe) form was characterized by a late formation of the main clinical symptoms of the disease: the first external signs of the disease became evident at 5–6 months of life, heart murmurs were heard at 7–8 months, at the same age hepatosplenomegaly was diagnosed. After six months of life, the child experienced a delay in psychomotor development. However, the children of the second group were capable of independent movement, phrasal speech with limited vocabulary and successful communication. These patients were distinguished by disinhibition, restlessness, quick exhaustion, and lack of concentration. The IQ of the second group of patients did not exceed 45–55 points, and life expectancy, as a rule, was no more than 15–20 years.

The residual activity of I2S in plasma varied from undetectable values to 21.6 nmol/4h/ml. No residual activity in dried blood spots was determined.

The most common nucleotide variants in this group of patients was splicing site substitution c.1122C>T.

Three novel missense variant have been found in these patients: c.283A>T; c.512G>A; [c.1411G>C;1418C>T]. According to the prediction of damaging effect, all these nucleotide variants were disease causing ones (Table 3). Two different nucleotide variants [c.1411G>C;1418C>T] had been detected in one patient. First, it was assumed that one of these nucleotide variants was a nonpathogenic polymorphic one. However, both substitutions had been predicted as nucleotide variants with a potential damaging effect on the protein. For this case, it would be interesting to perform mutagenesis in vitro studies to assess the pathogenic effect of each of the substitutions. Two novel small deletions c.118_120delCTT; p.Leu40del and c.1426_1437 del12 p.476_479delAsnSerAspLys result in only loss of some amino acids without frame shift.

The clinical phenotype of the patients from Group 2b (a mild ->  an intermediate form (rather mild)) was between phenotypes of patients from Group 2a and Group 3. The residual activity of I2S in plasma varied from undetectable values to 21.6 nmol/4h/ml. No residual activity in dried blood spots was determined.

The nucleotide variant c.253G>A has been found in five patients from Group 2b. Nucleotide variants c.587T>C and c.1204G>A have been detected twice. Two missense variants c.1028G>A; c.1035G>C, site splicing substitution c.241-9C>G and c.1438_1442delCCGAG; p.Pro480Phefs were novel ones.

The third group of probands with a mild form of the disease was characterized by even a later formation (2–4 years of life) of clinical symptoms. These patients were distinguished by higher body length, less severe changes in the musculoskeletal system and internal organs, a rare formation of hernias or their complete absence, normal intelligence, allowing probands to study in primary, secondary and even professionally oriented schools, successfully graduate and even work in their chosen field, often climbing the corporate ladder to senior level positions. Many of them successfully married and had a healthy offspring. The life expectancy of these patients was the highest and could reach 60 years and above.

The residual activity of I2S in plasma varied from undetectable values to 21.1 nmol/4 h/ml. No residual activity in dried blood spots was determined.

 Two variants (c.187A>G, p.1037C>T) were described elsewhere also in a patient with an attenuated form of the disease [24–27]. The four missense variants found in patients with a mild form of Hunter disease (c.236C>A; c.305T>G; c.593A>G; c.1019G>A) were described by authors in their first study of the Russian patient group with MPS II [28]. The nucleotide variants c.590C>T and c.1234G>T were first described in this study.

For all patients the values of the residual activity of I2S in plasma ranged from 0 to 21.6 nmol/4h/ml regardless of age and severity of the disease. For all patients, regardless of age and severity of the disease no residual activity in dried blood spots was determined. Thus, no correlation between the residual activity of enzyme and severity of the disease was observed (Table 4).

## Conclusion

In all groups of patients, a different type of nucleotide variant in the *IDS* gene has been found. It is assumed that the relationship of missense substitution with a severe form of Hunter syndrome, in some cases, can be explained by the pathological role of the replaced amino acid. On the other hand, two nonsense variants: c.1234G>T; p.Gly412Term and c.1438_1442delCCGAG; p.Pro480Phefs, despite the formation of the premature stop codon led to the development of a mild form or a mild -> intermediate form of Hunter syndrome. That suggests that a break in the amino acid chain at a certain position does not always cause several functional damages to the protein. Thus, in authors’ opinion, in order to understand the effect of missense and even nonsense substitutions, specific functional studies are required.

Several point variants (c.253G>A, c.257C>T, c.263G>A, c.263G>T, c.514C>T, c.998C>T, c.1327C>T, c.1402C>T; c.1403G>A, c.1122C>T), a small deletion c.596_599delAACA and recombination between intron 7 in the *IDS* and exon 3 in *IDS2* were detected more than twice in patients from different families. The frequency of these substitutions in the presented cohort of patients varies from 2 to 6%. Thus, these replacements could be relatively common. Probably these point variants that involved CpG sites of the *IDS* gene, the locus of c.596_599delAACA deletion and recombination region between the *IDS* and *IDS2* are mutagenesis hotspots in the *IDS* gene [18, 19].

The enzyme replacement therapy with Elaprase has become available in Russia since 2008. According to the Russian public health law, Elaprase was purchased by regional health authorities, which do not always afford the drug due to its high cost. Therefore, not all patients with Hunter syndrome received enzyme replacement therapy. Since 2019, federal authorities started purchasing Elaprase at the expense of the federal budget to provide all patients with mucopolysaccharidosis type II with this enzyme replacing drug.

In 2018, a second enzyme-replacing drug was registered in Russia for the treatment of patients with Hunter syndrome, called idursulfase beta (Hunterase). The drug was developed and successfully tested by Korean researchers.

To date, 113 patients with Hunter syndrome are receiving enzyme replacement therapy in Russia, including 16 adult patients. Enzyme replacement therapy undoubtedly improves the quality and life expectancy of patients with Hunter syndrome, especially patients with a mild form of the disease. In patients with severe (Group 1) and intermediate (Group 2a) forms of the disease, the improvement relates mainly to the state of internal organs (reduction in size liver and spleen, decreased left ventricular mass), increased weight and growth parameters, mainly body length, improved emotional tone, increased step test (if not independent movement of patients), and decreased renal excretion of heparan and dermatan.

No growth in intellectual development (IQ) was observed due to the inability of enzyme replacing drugs to penetrate the blood–brain barrier.

During treatment, patients with a mild form of the disease had more positive changes and a notable increase in tolerance to physical and mental stress.

## Data Availability

The datasets generated and analysed during the current study are available in the Human Gene Mutation Database www.hgmd.cf.ac.uk/ (information about nucleotide changes described before) and ClinVar at https://www.ncbi.nlm.nih.gov/clinvar/ (information about novel nucleotide changes), ClinVar accession numbers SCV001450592-SCV001450635 (see also Table 2). The information about the *IDS* gene sequencing is available at https://www.ncbi.nlm.nih.gov/genome/gdv/ (NC_000023.11).

## Abbreviations

DNA: Deoxyribonucleic acid
GAG: Glycosaminoglycans
I2S: Iduronate-2-sulfatase
MPS II: Mucopolysaccharidosis type II
